# Factors associated with the decision to terminate resuscitation early for adult in-hospital cardiac arrest: Influence of family in an East Asian society

**DOI:** 10.1371/journal.pone.0213168

**Published:** 2019-03-07

**Authors:** Chih-Hung Wang, Wei-Tien Chang, Chien-Hua Huang, Min-Shan Tsai, Ping-Hsun Yu, Yen-Wen Wu, Wen-Jone Chen

**Affiliations:** 1 Department of Emergency Medicine, National Taiwan University Hospital, Taipei, Taiwan; 2 Department of Emergency Medicine, College of Medicine, National Taiwan University, Taipei, Taiwan; 3 Department of Emergency Medicine, Taipei Hospital, Ministry of Health and Welfare, New Taipei City, Taiwan; 4 Department of Nuclear Medicine, Far Eastern Memorial Hospital, New Taipei City, Taiwan; 5 Division of Cardiology, Cardiovascular Medical Center, Far Eastern Memorial Hospital, New Taipei City, Taiwan; 6 National Yang-Ming University School of Medicine, Taipei, Taiwan; 7 Division of Cardiology, Department of Internal Medicine, National Taiwan University Hospital, Taipei, Taiwan; Azienda Ospedaliero Universitaria Careggi, ITALY

## Abstract

**Background:**

We attempted to identify factors associated with physicians’ decisions to terminate CPR and to explore the role of family in the decision-making process.

**Methods:**

We conducted a retrospective observational study in a single center in Taiwan. Patients who experienced in-hospital cardiac arrest (IHCA) between 2006 and 2014 were screened for study inclusion. Multivariate survival analysis was conducted to identify independent variables associated with IHCA outcomes using the Cox proportional hazards model.

**Results:**

A total of 1525 patients were included in the study. Family was present at the beginning of CPR during 722 (47.3%) resuscitation events. The median CPR duration was significantly shorter for patients with family present at the beginning of CPR than for those without family present (23.5 mins vs 30 min, *p* = 0.01). Some factors were associated with shorter time to termination of CPR, including arrest in an intensive care unit, Charlson comorbidity index score greater than 2, age older than 79 years, baseline evidence of motor, cognitive, or functional deficits, and vasopressors in place at time of arrest. After adjusting for confounding effects, family presence was associated with shorter time to termination of CPR (hazard ratio, 1.25; 95% confidence interval, 1.06–1.46; *p* = 0.008).

**Conclusion:**

Clinicians’ decisions concerning when to terminate CPR seemed to be based on outcome prognosticators. Family presence at the beginning of CPR was associated with shorter duration of CPR. Effective communication, along with outcome prediction tools, may avoid prolonged CPR efforts in an East Asian society.

## Introduction

In the United States, approximately 209,000 patients experience in-hospital cardiac arrest (IHCA) each year [1]. Despite ongoing efforts to improve the “chain of survival,” patient outcomes after IHCA remain poor. Approximately 24% of IHCA patients survive to hospital discharge; among these patients, roughly 14% experience significant neurological disability [1].

The most important treatment for patients experiencing cardiac arrest is cardiopulmonary resuscitation (CPR) but the optimal duration of CPR efforts is debated. Previous studies have reported that longer CPR duration was associated with worse outcomes for patients who experienced out-of-hospital cardiac arrest (OHCA) [2, 3]. Nonetheless, some subgroups of OHCA, such as patients with initial shockable rhythms [4], have been demonstrated to have higher resilience for favourable outcomes even after prolonged CPR. The minimum CPR duration before declaration of death for OHCA remains unclear since the no-flow time of each patient is different and the tolerability for low-flow time may vary [5].

Few studies have explored the associations between CPR duration and IHCA outcomes. Goldberger et al. [6] posited that the survival probability of IHCA would be higher with longer CPR attempts. Compared with OHCA, survival may be higher for IHCA because experienced healthcare staff and advanced treatments could be involved in the early phase of CPR. Owing to advanced resuscitation skills and therapeutics, it is difficult to establish an arbitrary time point to terminate CPR for IHCA. Van Walraven et al. [7] proposed a rule to terminate resuscitation efforts for IHCA: if the arrest was not witnessed, the initial rhythms were non-shockable, and return of spontaneous circulation (ROSC) did not occur within 10 minutes of initiation of CPR, the survival probability would be extremely low and discontinuation of resuscitation efforts should be considered. However, Chen et al. [8] demonstrated that, even when conventional CPR was continued for more than 10 minutes, extracorporeal CPR may still have benefits for some subgroups of patients experiencing IHCA.

Since CPR duration was associated with outcomes [6], it is important to identify factors associated with the decision to terminate CPR early for IHCA. Some patient-specific and hospital-specific factors have been reported to be associated with CPR duration for IHCA [9]. However, most of these observations were made in a Western society [9]. Factors influencing CPR duration may vary between East Asian and Western societies since cross-cultural perspectives about end-of-life care are different [10]. Specifically, compared with Western societies, East Asian societies may put more emphasis on family-centered end-of-life care [10]. Therefore, in the current analysis, we attempted to explore the influence of patient factors on CPR duration and investigate the association of family presence with the decision to terminate CPR.

## Materials and methods

### Setting

This retrospective cohort study was performed at National Taiwan University Hospital (NTUH), which is a tertiary medical center. NTUH has 2600 beds, including 220 beds in intensive care units (ICUs). This study was conducted in accordance with the Declaration of Helsinki amendments. The Research Ethics Committee of NTUH approved this study (reference number: 201706033RINA) and waived the requirement for informed consent before data collection.

According to hospital policy, a code team is activated when a cardiac arrest event occurs in the general wards. A code team consists of a senior resident, several junior residents, a respiratory therapist, a head nurse, and several ICU nurses. Each code team member is certified to provide basic and advanced life support according to current resuscitation guidelines. A code team is not mobilized for cardiac arrest events in the ICUs; instead, resuscitation is performed by the ICU staff where the event occurs and by staff from neighboring ICUs.

NTUH has no specific policy concerning whether family can or cannot be present during CPR. When IHCA occurs, the family is positioned away from the bedside to make space for the healthcare staff to perform CPR. The family is still able to hear sounds related to CPR, such as voices of healthcare staff and noises of chest compression machines. The decision to allow a family to witness the whole or part of the CPR process is made according to clinicians’ discretions. If the family is not present at the bedside when IHCA occurs, clinicians perform CPR immediately; the family is contacted by phone and the emergent condition of the patient is conveyed. The decision to terminate CPR attempts is made after consensus has been achieved between family members and clinicians. Such peri-arrest scenarios are documented in the medical records.

### Patients

Patients who experienced IHCA at NTUH between 2006 and 2014 were screened for study inclusion. Patients who met the following criteria were included in the study: (1) age 18 years or older, (2) documented absence of pulse with performance of chest compressions for at least 2 minutes, and (3) no documentation of a do-not-resuscitate order before arrest. If multiple cardiac arrest events occurred in a single patient during hospitalization(s), only the first event was examined and analyzed because the factors associated with the outcomes of further events could be quite different. Patients who experienced cardiac arrest events related to major trauma were excluded from the study due to heterogeneity in etiology.

### Data collection and outcome measures

The following information was recorded for each patient: age, sex, comorbidities [11], variables derived from the Utstein template [12], and critical interventions implemented at the time of cardiac arrest. Transient ROSC was defined as ROSC persisting less than 20 minutes; sustained ROSC was defined as ROSC lasting 20 minutes or longer without resumption of chest compressions. The time points of the first transient ROSC and sustained ROSC were recorded for analysis.

### Statistical analysis

Data were analyzed using R 3.3.1 software (R Foundation for Statistical Computing, Vienna, Austria). Categorical data were expressed as counts and proportions; continuous data were expressed as median and interquartile ranges. In the univariate analysis, categorical variables were examined using Fisher’s exact test and continuous variables were compared using Wilcoxon’s rank-sum test. A two-tailed *p*-value of 0.05 or lower was considered to indicate statistical significance.

Multivariate analysis was conducted to identify independent variables associated with CPR duration by fitting Cox’s proportional hazards models using the *survival* package and the *My*.*stepwise* package of R software. The independent variables included age, sex, comorbidities, Charlson comorbidity index (CCI) score, timing and location of arrest, witnessed and monitored status, initial arrest rhythms, presence or absence of family at the beginning of the CPR, critical care interventions in place at time of arrest, and presence of transient ROSC. Moreover, termination of CPR without sustained ROSC was the event of interest and all subjects were right-censored either at the time when sustained ROSC was achieved or at the 120 minutes from the start of chest compressions [9]. Kaplan–Meier plot was depicted to visualize the association between independent variables and CPR duration.

All available independent variables were considered in the regression analysis, regardless of whether they showed significant associations with CPR duration in the univariate analysis. The stepwise variable selection procedure (with iterations between the forward and backward steps) was applied to obtain the candidate Cox’s proportional hazards model. The significance levels for entry and to stay were established at 0.15 to avoid exclusion of potentially important variables. The final Cox’s proportional hazards model was identified by removing individual variables with *p*-values greater than 0.05 one at a time until all regression coefficients were significantly different from 0. We used generalized additive models [13] to examine potential nonlinear effects of continuous variables and to identify appropriate cut-off point(s), if necessary, for dichotomizing continuous variables during the variable selection procedure. We assessed the goodness-of-fit of the fitted Cox’s proportional hazards model using the concordance (i.e., *c* statistic) and adjusted generalized *R*^2^.

## Results

A total of 1538 adult patients at NTUH received chest compressions for at least 2 minutes between 2006 and 2014. Of these, 13 patients were excluded from this study because of trauma-related cardiac arrest. The remaining 1525 patients were enrolled in our study for further analysis.

The baseline characteristics, peri-cardiac arrest events, and resuscitation outcomes for all patients are presented in Tables 1 and 2. Overall, the median age of the patients was 67.0 years, and 927 (60.8%) of the patients were male. Family was present at the beginning of CPR during 722 (47.3%) resuscitation events. There was no significant difference in CCI scores between patients with and without family present at the beginning of CPR. A total of 698 (45.8%) IHCA events occurred in ICUs, and 730 (47.9%) events occurred in general wards. The majority (84.7%) of initial arrest rhythms were non-shockable rhythms, including pulseless electrical activity and asystole. The median CPR duration was 26 minutes, but the duration of resuscitation efforts was significantly shorter for patients with family present at the beginning of CPR. A total of 876 (57.4%) patients achieved sustained ROSC.

**Table 1:** Baseline characteristics of study patients stratified by the presence of family members at initiation of cardiopulmonary resuscitation efforts.

Variables	All patients (n = 1525)	Family present at the beginning of CPR^b^ (n = 722)	Family absent at the beginning of CPR (n = 803)	*p*-value
Age (year), median (IQR^a^)	67.0 (54.7–78.2)	65.0 (53.2–76.4)	68.9 (56.2–80.3)	<0.001
Male, n (%)	927 (60.8)	447 (61.9)	480 (59.8)	0.40
Comorbidities, n (%)				
Heart failure, this admission	285 (18.7)	127 (17.6)	158 (19.7)	0.32
Heart failure, prior admission	240 (15.7)	101 (14.0)	139 (17.3)	0.08
Myocardial infarction, this admission	195 (12.8)	75 (10.4)	120 (14.9)	0.009
Myocardial infarction, prior admission	62 (4.1)	26 (3.6)	36 (4.5)	0.44
Arrhythmia	264 (17.3)	119 (16.5)	145 (18.1)	0.46
Hypotension	362 (23.7)	169 (23.4)	193 (24.0)	0.81
Respiratory insufficiency	1108 (72.7)	501 (69.4)	607 (75.6)	0.007
Renal insufficiency	631 (41.4)	281 (38.9)	350 (43.6)	0.07
Hepatic insufficiency	279 (18.3)	147 (20.4)	132 (16.4)	0.05
Metabolic or electrolyte abnormality	257 (16.9)	127 (17.6)	130 (16.2)	0.49
Diabetes mellitus	493 (32.3)	227 (31.4)	266 (33.1)	0.51
Chronic obstructive pulmonary disease	78 (5.1)	32 (4.4)	46 (5.7)	0.29
Baseline evidence of motor, cognitive, or functional deficits	460 (30.2)	219 (30.3)	241 (30.0)	0.91
Acute stroke	70 (4.6)	32 (4.4)	38 (4.7)	0.81
Favourable neurological status 24 h before cardiac arrest	652 (42.8)	355 (49.2)	297 (37.0)	<0.001
Bacteraemia	130 (8.5)	64 (8.9)	66 (8.2)	0.71
Metastatic cancer or any blood borne malignancy	344 (22.6)	177 (24.5)	167 (20.8)	0.08
Charlson comorbidity index score, median (IQR); mean (SD^c^)	2 (1–4)	2 (1–4); 2.9 (2.2)	2 (1–4); 2.8 (2.1)	0.41

^a^IQR, interquartile range.

^b^CPR, cardiopulmonary resuscitation.

^c^SD, standard deviation

**Table 2:** Features, interventions, and outcomes of cardiac arrest events stratified by the presence of family members at initiation of cardiopulmonary resuscitation efforts.

Variables	All patients (n = 1525)	Family present at the beginning of CPR (n = 722)	Family absent at the beginning of CPR (n = 803)	*p*-value
Arrest at night, n (%)	515 (33.8)	218 (30.2)	297 (37.0)	0.006
Arrest on weekend, n (%)	435 (28.5)	212 (29.4)	223 (27.8)	0.50
Arrest location, n (%)				<0.001
Intensive care unit	698 (45.8)	234 (32.4)	464 (57.8)	
General ward	730 (47.9)	424 (58.7)	306 (38.1)	
Others	97 (6.4)	64 (8.9)	33 (4.1)	
Witnessed arrest, n (%)	1086 (71.2)	480 (66.5)	606 (75.5)	<0.001
Monitored status, n (%)	939 (61.6)	393 (54.4)	546 (68.0)	<0.001
Shockable rhythm, n (%)	233 (15.3)	98 (13.6)	135 (16.8)	0.09
Critical care interventions in place at time of arrest, n (%)				
Mechanical ventilation	328 (21.5)	139 (19.3)	189 (23.5)	0.05
Antiarrhythmics	162 (10.6)	58 (8.0)	104 (13.0)	0.002
Vasopressors	649 (42.6)	251 (34.8)	398 (49.6)	<0.001
Dialysis	118 (7.7)	53 (7.3)	65 (8.1)	0.63
Pulmonary artery catheter	14 (0.9)	6 (0.8)	8 (1.0)	0.79
Intra-aortic balloon pumping	15 (1.0)	8 (1.1)	7 (0.9)	0.80
CPR^a^ duration (min), median (IQR^b^)	26 (11–45)	23.5 (11–42)	30 (12–47)	0.01
Transient ROSC,^c^ n (%)	331 (21.7)	146 (20.2)	185 (23.0)	0.19
Sustained ROSC, n (%)	876 (57.4)	433 (60.0)	443 (55.2)	0.06

^a^CPR, cardiopulmonary resuscitation

^b^IQR, interquartile range

^c^ROSC, return of spontaneous circulation

**Fig 1:**
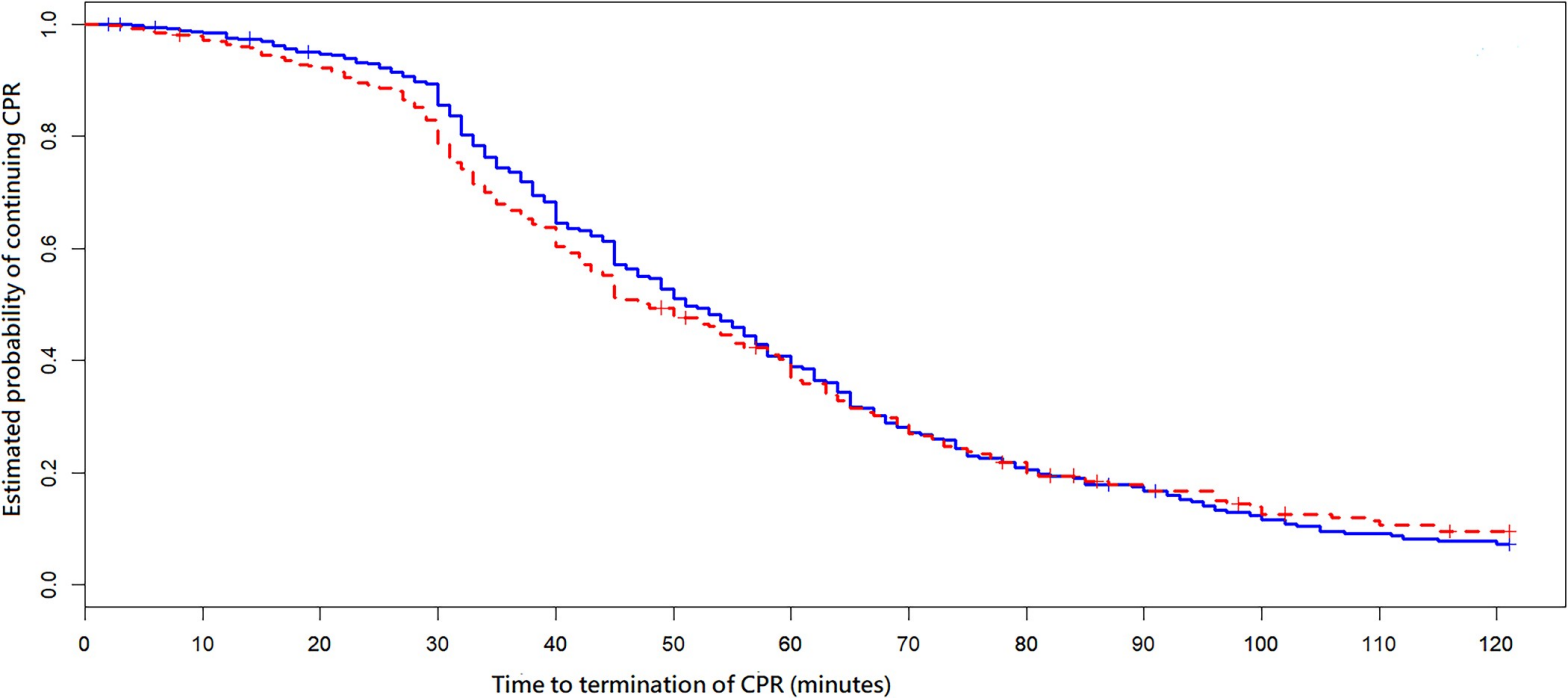
Kaplan–Meier plots depicting curves of time to termination of cardiopulmonary resuscitation. CPR, cardiopulmonary resuscitation. Dotted line: Family present at the beginning of CPR. Solid line: Family absent at the beginning of CPR.

All independent variables listed in Tables 1 and 2 were used in the multivariate survival analysis for variable selection. The hazard ratios (HRs) of factors that were significantly associated with outcomes are presented in Table 3. Several factors were noted to be associated with shorter time to terminate CPR efforts, including arrest at ICU (HR, 1.79; 95% confidence interval [CI], 1.48–2.17; *p* < 0.001); CCI score > 2 (HR, 1.52; 95% CI, 1.28–1.80; *p* < 0.001); age older than 79 years (HR, 1.46; 95% CI, 1.21–1.77; *p* < 0.001); family present at the beginning of CPR (HR, 1.25; 95% CI, 1.06–1.46; *p* = 0.008); baseline evidence of motor, cognitive, or functional deficits (HR, 1.24; 95% CI, 1.04–1.48; *p* = 0.02); and vasopressors in place at time of arrest (HR, 1.21; 95% CI, 1.01–1.46; *p* = 0.04). Fig 1 demonstrated the Kaplan–Meier plot based on the variable of family present at the beginning of CPR.

**Table 3:** Multivariate survival analysis for termination of cardiopulmonary resuscitation efforts using a Cox’s proportional hazards model.

Independent variable^a^	Hazard ratio	95% confidence interval	*p*-value
Arrest in intensive care unit	1.79	1.48–2.17	<0.001
Transient ROSC^b^	0.58	0.48–0.70	<0.001
Charlson comorbidity index score > 2	1.52	1.28–1.80	<0.001
Age older than 79 years	1.46	1.21–1.77	<0.001
Family present at the beginning of CPR^c^	1.40	1.18–1.65	<0.001
Arrest at other locations	0.41	0.24–0.70	0.001
Arrhythmia	0.69	0.54–0.87	0.002
Diabetes mellitus	0.75	0.62–0.91	0.003
Chronic obstructive pulmonary disease	0.63	0.43–0.91	0.02
Baseline evidence of motor, cognitive, or functional deficits	1.24	1.04–1.48	0.02
Myocardial infarction, this admission	0.73	0.56–0.95	0.02
Vasopressors in place at time of arrest	1.21	1.01–1.46	0.04

Goodness-of-fit assessment: concordance = 0.68, adjusted generalised *R*^*2*^ = 0.1.

^a^ The display of independent variables is arranged by the order of *p*-value.

^b^ ROSC, return of spontaneous circulation.

^c^ CPR, cardiopulmonary resuscitation

## Discussion

### Main findings

In this retrospective study, several factors were noted to be associated with shorter time to terminate CPR for adult IHCA. Most of these factors were associated with comorbidities or illness statuses of the patients, which reflected that the decision to terminate CPR early may be based on the predicted prognosis of IHCA. Also, family presence at the beginning of CPR was significantly associated with shorter duration to terminate CPR, implying that effective communication between clinicians and family members during resuscitation efforts may reduce CPR duration for IHCA in an East Asian society.

### Factors associated with CPR duration for IHCA

Khan et al. [9] reported that younger age was associated with longer CPR duration in patients who did not experience ROSC after IHCA. In the current study, we noted that age older than 79 years was associated with shorter duration to terminate CPR. This age-based difference in CPR efforts may be caused by perceived poor outcomes for the elderly following cardiac arrest. However, Chan et al. [14] indicated that nearly 60% of IHCA survivors aged 65 years or older were alive at 1 year, and, among this group, the rate of 3-year survival did not differ significantly from that of patients with heart failure. Therefore, it appears that age-based decisions to terminate CPR may not be justified.

In addition to age, other prognostic factors were noted to influence duration of CPR attempts. Arrest in ICU; CCI score > 2; baseline evidence of motor, cognitive, or functional deficits; and vasopressors in place at time of arrest were associated with shorter duration of CPR. Clinicians seemed to shorten CPR attempts for patients with unfavourable prognostic factors. Previous studies [15, 16] demonstrated that CCI score > 2 was associated with worse OHCA outcomes. Although CCI score was calculated retrospectively, our result basically meant that clinicians might terminate CPR earlier in patients with more comorbidities, which was recorded in the admission note. Bradley et al [17] demonstrated that CPR duration for IHCA was generally consistent with the predicted outcome: that is, longer duration of resuscitation efforts for higher predicted survival and shorter duration for lower predicted survival. However, Bradley et al. [17] also reported that approximately 40.4% of patients with predicted poor outcomes received longer-than-the-median duration of CPR and 31.9% of patients with predicted favourable outcomes received shorter-than-the-median duration [17]. This discordance suggested that the prognosticator-based estimation of minimum CPR duration may not be accurate and other unmeasured confounders may be involved in the clinical judgments regarding CPR duration.

### Influence of family presence on CPR duration

Jabre et al. [18] reported that family presence during CPR for OHCA was associated with positive psychological effects and did not interfere with medical interventions, such as CPR duration. They reported median CPR durations of 30 minutes for patients with and without family present [18]. However, these reported CPR durations included both survivors and non-survivors [18]. Accordingly, it was uncertain whether family presence interfered with the decision to terminate CPR for non-survivors. In an IHCA study, Goldberger et al. [19] noted a trend toward shorter CPR duration for non-survivors in hospitals allowing for family presence during CPR.

Our analysis demonstrated that family presence at the beginning of CPR was significantly associated with shorter time to terminate resuscitation efforts. In studies of end-of-life decision-making, surrogate decision-makers reported a desire to receive early communication from physicians and to be prepared gradually for poor outcomes [20]. When family is present at the beginning of CPR, it may be easier for clinicians to achieve consensus with family concerning when to terminate CPR. This difference from previous observations [18, 19] may be explained by racial and ethnic differences in attitudes toward end-of-life care [21, 22]. In Taiwanese society, there is a prevalent notion that “a bad life is better than a good death [10].” Therefore, family who does not realize how violent CPR is may request that clinicians extend CPR as long as the patient’s life could potentially be saved, even if the chances of recovering favourable neurological status are dismal, i.e. the chances of sustaining a bad life are high. Family who is present at the beginning of CPR may be more likely to realize the true conditions of CPR by directly witnessing the resuscitation scene or indirectly hearing the noises generated during resuscitation efforts; accordingly, the family may be more willing to discontinue CPR, if appropriate.

When substantial medical resources are focused on a single patient, the level of care might be inadequate for other patients [23, 24]. Prolonged CPR might not only cause the patient to suffer but also endanger other hospitalized patients [23, 24]. To avoid prolonged CPR duration, more accurate information should be provided to family to assist in determining when to terminate CPR, even if family members are not present at the beginning of CPR.

### Prediction rules to assist in communication with family

Jones et al. [25] found that fewer than 50% of medical students, residents, and attending physicians were able to accurately assess survival probability after IHCA. A previous study revealed that clinicians may adjust the CPR duration for OHCA patients according to their subjective predictions of futility [26]. This practice may make the observed unfavourable outcome for patients with poor prognostic indicators a self-fulfilling prophecy [26]. Therefore, family may be concerned that a patient did not receive the appropriate resuscitation efforts and worry that resuscitation efforts were terminated prematurely. This concern may be intensified if the family was not present at the beginning of CPR and did not have immediate access to the resuscitation scene. Moreover, the family may have a falsely high expectation of the long-term outcomes following IHCA [27] and, therefore, request that CPR be extended as long as possible. Nevertheless, prolonged CPR may not necessarily be aligned with a patient’s preferences. Effective communication with accurate prognostic information may help close this perception gap.

Our previous study [28] demonstrated that serum lactate level measured during CPR was associated with survival outcomes. A lactate level threshold of 9 mmol/L may be used as a reference value to identify patients with different survival probabilities and determine the optimal CPR durations. Even if IHCA patients were successfully resuscitated, the Cardiac Arrest Survival Post-resuscitation In-hospital [CASPRI] score could be used to provide an estimated probability of favourable neurological outcomes after ROSC [29, 30]. As shown in the current analysis, transient ROSC was associated with longer time to termination of CPR. By using the CASPRI score, it may be easier for family and clinicians to achieve consensus concerning the management strategy when patients experience re-arrest following transient ROSC [29]. This approach may help shorten prolonged CPR efforts since re-arrest is associated with decreased survival [31].

In summary, we identified several factors associated with the decision to terminate CPR efforts early. Most of these factors related to patient comorbidities or illness status. Family presence also significantly influenced this decision-making process, which has not been reported in other studies. The influence of family on CPR duration may be explained by the filial piety and family-centered end-of-life care stressed by Confucianism, which is unique to East Asian societies [10]. Further, family members may have very different definitions of medical futility than healthcare staff. With the assistance of prognostic tools, clinicians may inform the family of a more accurate estimated probability for favourable outcomes after IHCA. Prolonged resuscitation efforts might be avoided, which would be in the best interest of the patient.

### Study limitations

This study has several limitations. First, this was an observational study that can only establish an association, rather than a causal relationship, between independent and dependent variables. For family to determine on terminating CPR, the psychological mechanisms underlying this decision are quite complex and could be not verified through a retrospective design. Second, this study was conducted in a single medical center in Taiwan. The results may not be generalized to other racial or ethnic groups. Also, we did not include patients experiencing traumatic cardiac arrest in analysis because the epidemiological data were quite different between traumatic and medical cardiac arrest [32]. The conclusions of the current study may not be applied to patients experiencing traumatic cardiac arrest. Third, our study design was retrospective, so we do not actually know whether family witnessed CPR efforts. However, we assumed that family who only heard the sounds generated during CPR, especially the noise made by the piston-driven chest compression machine, would feel the same way as family who were able to witness CPR efforts entirely.

## Conclusions

Clinicians’ decisions concerning when to terminate CPR seemed to be based on outcome prognosticators. Family presence at the beginning of CPR was associated with shorter duration of CPR. Effective communication, along with outcome prediction tools, may avoid prolonged CPR efforts in an East Asian society.

## Supporting information

S1 Dataset Raw data used in statistical analysis. (XLSX)
